# Impact of long-term steroid therapy on epicardial and pericardial fat deposition: a cardiac MRI study

**DOI:** 10.1186/s12933-015-0289-x

**Published:** 2015-09-30

**Authors:** Daniel Kitterer, Joerg Latus, Joerg Henes, Stefan Birkmeier, Maik Backes, Niko Braun, Udo Sechtem, M. Dominik Alscher, Heiko Mahrholdt, Simon Greulich

**Affiliations:** Division of Nephrology, Department of Internal Medicine, Robert-Bosch-Medical Center, Auerbachstrasse 110, 70376 Stuttgart, Germany; Division of Rheumatology, Department of Internal Medicine II, Universitätsklinik Tübingen, Tübingen, Germany; Department of Cardiology, Robert-Bosch-Medical Center, Auerbachstrasse 110, 70376 Stuttgart, Germany; Department of Radiology, Robert-Bosch-Medical Center, Auerbachstrasse 110, 70376 Stuttgart, Germany

**Keywords:** Cardiac MRI, Epicardial fat, Pericardial fat, Steroid therapy, Rheumatic disorder

## Abstract

**Background:**

Increased cardiac fat has been identified as a risk factor for coronary artery disease. Metabolic syndrome is associated with increased cardiac fat deposition. Steroids are known to imitate some effects of metabolic syndrome and are frequently used in patients with rheumatic disorders. Primary aim was to evaluate the impact of long-term steroid use on cardiac fat deposition in patients with rheumatic disorders. In addition, we sought to investigate if this effect might be dose-dependent.

**Methods:**

Patients were enrolled as follows: (1) rheumatic disorder; and (2) long-term steroid therapy, and (3) underwent cardiovascular magnetic resonance (CMR) imaging. Patients were stratified in a high-dose (>7.5 mg prednisone equivalent/day for at least 6 months) and a low-dose steroid group (<7.5 mg prednisone equivalent/day) and compared to steroid-naïve controls without rheumatic disorders.

**Results:**

122 patients were included (n = 61 steroid patients, n = 61 controls). N = 36 were classified as high-dose, n = 25 as low-dose steroid group. Steroid patients showed larger epicardial 5.7 [3.5–9.1] cm^2^ and pericardial 13.0 [6.1–26.8] cm^2^ areas of fat than controls 4.2 [1.3–5.8] cm^2^/6.4 [1.6–15.4] cm^2^, p < 0.001, p < 0.01, respectively. High-dose steroid patients had more epi- and pericardial fat both than controls: 7.2 [4.2–11.1] cm^2^ vs. 4.4 [1.0-6.0] cm^2^, p < 0.001; 18.6 [8.9–38.2] cm^2^ vs. 10.7 [4.7–26.8] cm^2^, p < 0.05, and patients in the low-dose steroid group (p < 0.01, p < 0.001, respectively).

**Conclusion:**

The present data suggest increased cardiac fat deposition in steroid-treated patients with rheumatic disorders. Furthermore, this accumulation of cardiac fat seems to be dose-dependent, pointing towards a cumulative effect of steroids.

## Background

Coronary artery disease (CAD) is the leading cause of death in the western world [1]. Current clinical practice guidelines recommend strict control of established cardiovascular risk factors such as diabetes, arterial hypertension, obesity and dyslipidemia [2]. Beside these traditional cardiovascular risk factors, there is growing interest in new potential cardiovascular risk factors, which may help improving patients’ treatment and/or prognosis. One of these potential new risk factors is cardiac adipose tissue, since recent studies reported that increased cardiac adipose tissue in the setting of metabolic syndrome [3] (and in particular accumulation of epicardial adipose tissue) is associated with the presence of coronary artery disease [4–6], as well as with cardiovascular adverse events [7, 8].

Steroid medication is known to imitate many effects of the metabolic syndrome [9]. However, the impact of a long-term steroid therapy on myocardial adipose tissue deposition in a clinical setting has not been investigated so far. This is of special interest in the large group of patients with different rheumatic disorders, since these patients are often dependent on the long-term use of steroids.

Thus, our primary aim was to evaluate the impact of long-term steroid therapy on cardiac fat deposition in patients with rheumatic disorders. In addition, we sought to investigate if this effect might be dose-dependent.

## Methods

### Patient population

Sixty-one consecutive patients presenting at our institution between October 2012 and November 2013 were prospectively enrolled if they fulfilled the following criteria: (1) underlying rheumatic disorder; and (2) long-term steroid medication; and (3) successfully underwent cardiovascular magnetic resonance (CMR) imaging. Patients´ medical history was reviewed in detail regarding dosage and duration of steroid therapy, and clustered in a high-dose and a low-dose steroid group (definitions see below). Exclusion criteria were contraindications for CMR (e.g. pregnancy, pacemaker/implantable cardioverter defibrillator (ICD), glomerular filtration rate <30 ml/min., previous adverse reactions to gadolinium, cochlea implant). Patients gave written informed consent according to the Declaration of Helsinki prior to inclusion in the study. The data collection and the study have been approved by the ethics committee of the University of Tübingen (527/2012B02).

### Control group

Sixty-one age-, sex-, and body mass index (BMI) matched patients with no rheumatic disorder, and who never had been on steroids served as control group. Due to the paired matched character of the control group, these controls were further divided in two groups to match (1) the high-dose steroid group, and (2) the low-dose steroid group. Baseline characteristics of the patients with steroid therapy and the paired matched control groups can be viewed in Table 1.

**Table 1:** Baseline patient characteristics

Variable	Steroid-treated patients	Steroid-naïve controls	p
n	61	61	–
Age (years)	54 ± 16	54 ± 16	–
Female	43 (70.5)	43 (70.5)	–
BMI, kg/m^2^	27 ± 6	27 ± 6	–
LVEF, %	62 [60–67]	66 [59–69]	0.16
LVEDV, ml	121 [108–136]	117 [102–140]	0.99
LVESV, ml	44 [35–56]	42 [33–5]	0.74
Epicardial fat, cm^2^	5.7 [3.5–9.1]	4.2 [1.3–5.8]	<0.001
Pericardial fat, cm^2^	13.0 [6.1–26.8]	6.4 [1.6–15.4]	<0.01
CAD	9 (14.8)	0	<0.01
Arterial hypertension	33 (54.1)	23 (37.7)	0.10
Systolic blood pressure, mmHg	127 ± 20	118 ± 16	<0.05
Diastolic blood pressure, mmHg	74 ± 10	71 ± 8	0.18
Diabetes	10 (16.4)	1 (0.2)	<0.01
Hypercholesterolemia	10 (16.4)	13 (21.3)	0.44
Total cholesterol, mmol/l	5.34 ± 1.24	5.18 ± 0.95	0.32
LDL cholesterol, mmol/l	3.26 ± 1.02	3.07 ± 0.95	0.41
HDL cholesterol, mmol/l	1.51 ± 0.49	1.43 ± 0.52	0.51
Triglycerides, mmol/l	4.56 ± 3.38	3.56 ± 1.77	0.05
Smoker	12 (19.7)	14 (23.0)	0.83
Family history of CAD	27 (44.3)	13 (21.3)	<0.05
Duration steroid therapy, months	30 [8–93]	–	–
Daily steroid dose at inclusion, mg	10 [5–30]	–	–
Metabolic syndrome	2 (3.2)	0	0.50
ANCA pos. vasculitis^a^	21 (34.4)	–	–
Other vasculitis^b^	6 (9.8)	–	–
Collagenosis^c^	20 (32.8)	–	–
RA	10 (16.4)	–	–
Sarcoidosis	2 (3.3)	–	–
Others^d^	2 (3.3)	–	–

Values are mean ± SD, median [IQR], n (%)

*BMI* body mass index, LVEF left ventricular ejection fraction, *LVEDV* left ventricular end-diastolic volume, *LVESV* left ventricular end-systolic volume, *CAD* coronary artery disease, *LDL* low-density lipoprotein, *HDL* high-density lipoprotein, *ANCA* anti-neutrophil cytoplasmic antibody, *RA* rheumatoid arthritis

^a^ANCA pos. vasculitis: granulomatosis with polyangiitis, eosinophilic granulomatosis with polyangiitis, microscopic polyangiitis

^b^Other vasculitis: IgA vasculitis, Kawasaki disease, Takayasu’s Arteritis, giant cell arteritis

^c^Collagenosis: systemic lupus erythematosus, overlap syndrome, systemic sclerosis, sjogrens syndrome

^d^Others: Behçet’s disease, sarcoidosis

### CMR protocol

Electrocardiogram (ECG) gated CMR imaging was performed in breath-hold using a 1.5T Aera (Siemens-Healthcare, Germany) in line with recommendations of the Society of Cardiac Magnetic Resonance (SCMR) and the European Society of Cardiology (ESC) Working Group EuroCMR, respectively [10]. Cine short axis CMR images were prescribed every 10 mm (slice thickness 6 mm) from base to apex. In-plane resolution was typically 1.2 × 1.8 mm. Cine CMR was performed using a steady-state–free-precession-sequence.

### CMR analysis

Scans were analyzed by consensus of two experienced observers, who were blinded to patient identity and clinical information.

Cine images were evaluated as described previously [11]. In brief, endocardial and epicardial borders were outlined on the short axis cine images. Volumes and ejection fraction were derived by summation of epicardial and endocardial contours.

**Fig. 1:**
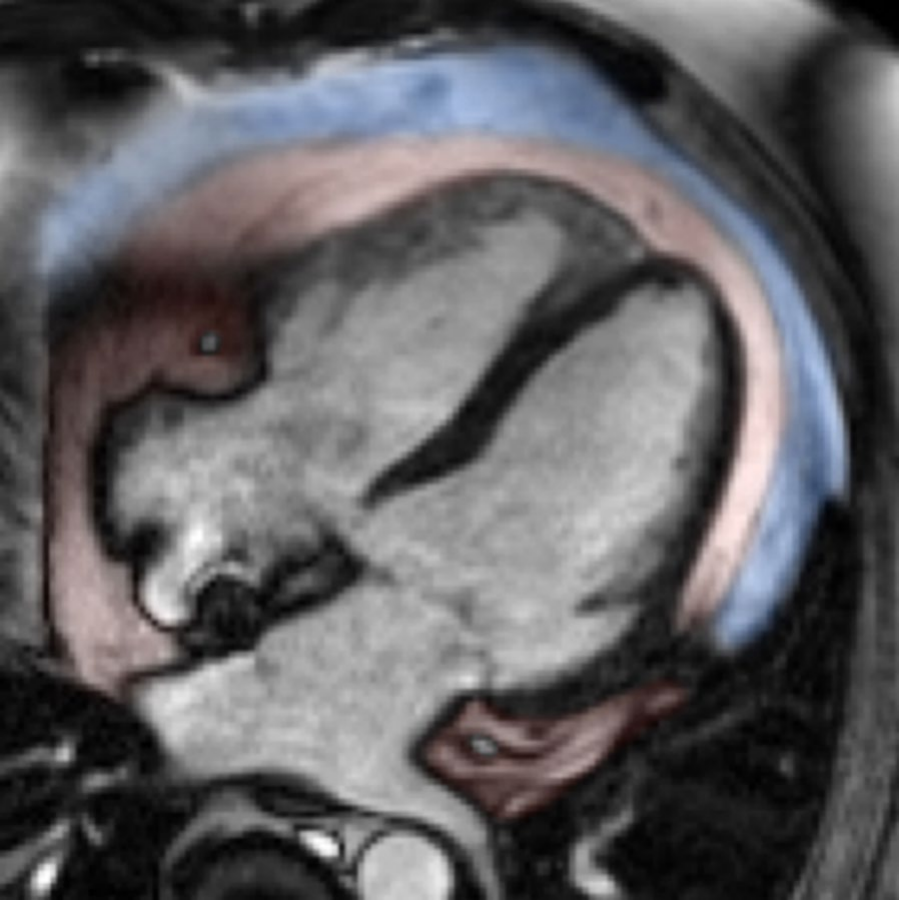
Determination of epicardial and pericardial adipose tissue: Epicardial (shown in *red*) and pericardial (shown in *blue*) contours were drawn in the end-diastolic image in a CMR 4-chamber view

A single cine four-chamber view of each patient was used for quantification (in cm^2^) of epicardial and pericardial fat layer (definitions see below), as described elsewhere [3, 12]. In brief, after careful examination of all phases of the cine four-chamber view image, epicardial and pericardial fat layers were outlined in the end-diastolic image with commercially available Siemens Argus software (Siemens-Healthcare, Germany), also see Fig. 1. Intra- and inter-observer variability was evaluated in 30 patients (15 steroid-treated patients, 15 steroid-naïve controls) on separate occasions.

### Definitions and variables

High-dose steroid group: Patients with an intake of >7.5 mg prednisone equivalent per day (Cushing threshold) for at least 6 months before CMR exam [13].

Low-dose steroid group: Patients with an intake of <7.5 mg prednisone equivalent per day for at least 6 months before CMR exam [13].

Long-term steroid use: Use of prednisone (or equivalent) daily, minimum during the past 6 months before CMR exam [13].

All glucocorticoid preparations were calculated to prednisone equivalent doses as described elsewhere [14].

Epicardial fat: Adipose tissue layer located between the myocardium and the visceral pericardium [3].

Pericardial fat: Intrathoracic adipose tissue located outside the parietal pericardium [12].

Body mass index (BMI): Weight (in kilograms) divided by the square of the height (in meters).

CAD: recognized or unrecognized myocardial infarction, or CAD with >20 % luminal diameter stenosis in at least one major coronary artery or its branches [15].

Arterial hypertension: Systolic blood pressure above 140 mmHg, diastolic blood pressure above 90 mmHg, or antihypertensive treatment [8].

Diabetes: Fasting plasma glucose level of ≥126 mg/dl or treatment with either insulin or a hypoglycemic agent.

Hypercholesterolemia: Total cholesterol level >200 mg/dl or use of cholesterol-lowering medication.

Smoker: Current or ever smokers.

Family history of CAD: Symptomatic CAD in a male first-degree relative before age 55, or a female first-degree relative before age 65 [16].

Metabolic syndrome: Patients had to fulfill the criteria of the International Diabetes Federation, which were described in detail previously [17].

### Statistical analysis

All continuous variables were tested for normality using the Kolmogorov–Smirnov test. Normally distributed continuous variables were expressed as means (with standard deviation) and skewed variables were presented as medians (with quartiles). Comparisons between groups were made using the Mann–Whitney U test or the Fisher’s exact test, as appropriate. Comparisons between paired groups were made using the Wilcoxon test. Intra- and inter-observer variability was assessed via intra-class correlation coefficients (ICC). Absolute agreement ICCs were calculated by a two-way mixed model for single measures. P values (two-tailed) of <0.05 were considered significant. All statistical analyses were performed using the GraphPad Prism statistical software package (GraphPad, San Diego, CA, USA) and SPSS Statistics version 23 (IBM Corporation, Armonk, NY, USA), respectively.

## Results

### Patient population

In total 122 patients were included in the final analysis: n = 61 patients received long-term steroid therapy due to underlying rheumatic disorder, n = 61 patients served as an age, gender and BMI-matched steroid-naïve control group. At inclusion, patients were 54 ± 16 years of age, predominantly female (71 %), with a mean BMI of 27 ± 6 kg/m^2^. Median disease duration was 4.7 [0.8–8.2] years. Functional CMR parameters did not differ significantly between both groups. However, patients on steroid therapy showed larger epicardial 5.7 [3.5–9.1] cm^2^, as well as pericardial 13.0 [6.1–26.8] cm^2^ fat areas compared to paired matched controls (4.2 [1.3–5.8] cm^2^/6.4 [1.6–15.4] cm^2^, p < 0.001, p < 0.01, respectively). Additional clinical characteristics can be viewed in Table 1.

In the steroid group, duration of steroid therapy was 30 [8–93] months with a median daily prednisone dose at inclusion of 10 [5–30] mg. Of note, the prevalence of metabolic syndrome did not differ significantly between the steroid group and controls; two out of 61 patients in the steroid group fulfilled criteria of metabolic syndrome, no patient in the control group, p = 0.5.

The majority of steroid-treated patients presented with ANCA positive vasculitis (34 %), followed by patients with collagenosis (33 %), and patients with rheumatoid arthritis (16 %).

### Low-dose steroid group vs. high-dose steroid group

Clinical data of the steroid population divided in low-dose and high-dose steroid groups are displayed in Table 2. Twenty-five (out of 61) patients were in the low-dose steroid group, 36 (out of 61) patients were in the high-dose steroid group. BMI was higher in the high-dose steroid (28 ± 6 kg/m^2^) compared to the low-dose steroid group (25 ± 5 kg/m^2^, p < 0.05). Functional CMR parameters were similar between both groups. More patients in the high-dose steroid group (n = 7) had prevalent CAD than in the low-dose steroid group (n = 2; p = 0.29). Traditional cardiovascular risk factors did not differ significantly between both groups. Metabolic syndrome was present in two patients of the high-dose steroid group, no patient in the low-dose steroid group fulfilled criteria of metabolic syndrome, p = 0.51.

**Table 2:** Clinical data of steroid population

Variable	Low-dose steroid group	High-dose steroid group	p
n	25	36	–
Age (years ± SD)	52 ± 19	55 ± 14	0.60
Female	18 (72.0)	25 (69.4)	–
BMI, kg/m^2^	25 ± 5	28 ± 6	<0.05
LVEF, %	62 [61–71]	62 [56–65]	0.19
LVEDV, ml	120 [100–136]	121 [108–137]	0.64
LVESV, ml	42 [30–56]	45 [37–57]	0.22
Epicardial fat, cm^2^	4.7 [2.1–7.5]	7.2 [4.2–11.1]	<0.01
Pericardial fat, cm^2^	8.3 [2.3–18.7]	18.6 [8.9–38.2]	<0.001
CAD	2/25 (8.0)	7/36 (19.4)	0.29
Arterial hypertension	11 (44.0)	22 (61.1)	0.09
Systolic blood pressure, mmHg	123 ± 16	128 ± 21	0.23
Diastolic blood pressure, mmHg	74 ± 9	73 ± 10	0.81
Diabetes	3 (12.0)	7 (19.4)	0.51
Hypercholesterolemia	2 (8.0)	8 (22.2)	0.18
Total cholesterol, mmol/l	5.2 ± 3.12	5.37 ± 1.19	0.64
LDL cholesterol, mmol/l	3.2 ± 1.11	3.25 ± 0.98	0.88
HDL cholesterol, mmol/l	1.48 ± 0.52	1.52 ± 0.48	0.77
Triglycerides, mmol/l	4.46 ± 3.28	4.53 ± 3.57	0.95
Smoker	5 (20.0)	7 (19.4)	1.00
Positive family history of CAD	12 (48.0)	15 (41.7)	0.79
Daily steroid dose at inclusion, mg	5.0 [3.8–7.5]	15 [10–28]	0.46
Metabolic syndrome	0	2 (5.5)	0.51

Values are mean ± SD, median [IQR], n (%); abbreviations see Table 1

### Epicardial and pericardial fat

**Fig. 2:**
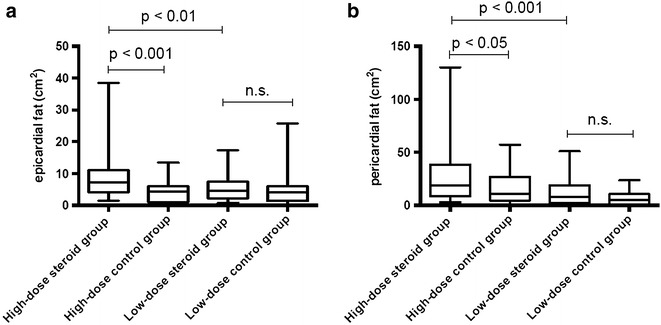
Values of epicardial and pericardial fat deposition in steroid-treated patients and matched steroid-naïve controls. **a** The high-dose steroid group (>7.5 mg prednisone equivalent daily) showed significant higher amounts of epicardial fat than the low-dose steroid group (<7.5 mg prednisone equivalent daily) and the age, sex and BMI matched steroid-naïve controls. In addition, the amount of epicardial fat was not significant different between low-dose steroid patients and the control group. **b** Likewise, these results could be confirmed for pericardial fat

Focusing on patients with high-dose steroid therapy (>7.5 mg prednisone equivalent daily during the past 6 months) revealed, that those patients had significantly more epicardial fat compared to matched steroid-naïve controls: 7.2 [4.2-11.1] cm^2^ vs. 4.4 [1.0–6.0] cm^2^, p < 0.001. Furthermore, patients on high-dose steroid therapy also had significantly more epicardial fat compared to patients on low-dose steroid therapy (<7.5 mg prednisone equivalent daily during the past 6 months): 7.2 [4.3–11] cm^2^ vs. 4.7 [2.1–7.5] cm^2^, p < 0.01, see Fig. 2.

Among patients on high-dose steroid therapy, CMR revealed significantly more pericardial fat compared to steroid-naïve controls: 18.6 [8.9–38.2] cm^2^ vs. 10.7 [4.7–26.8] cm^2^, p < 0.05. Additionally, patients on high-dose steroid therapy had significantly more pericardial fat compared to patients on low-dose steroid therapy: 18.6 [8.9–38.2] cm^2^ vs. 8.3 [2.3–18.7] cm^2^, p < 0.001.

No significant differences in epicardial and pericardial fat deposition could be detected between patients on low-dose steroid therapy and paired matched controls, see also Fig. 2.

Intra-observer reproducibility was high with an ICC of 0.97 for epicardial fat and 0.98 for pericardial fat assessment, respectively. Inter-observer reproducibility showed an ICC of 0.95 for epicardial and 0.96 for pericardial fat, respectively.

### Cardiac fat and BMI

**Fig. 3:**
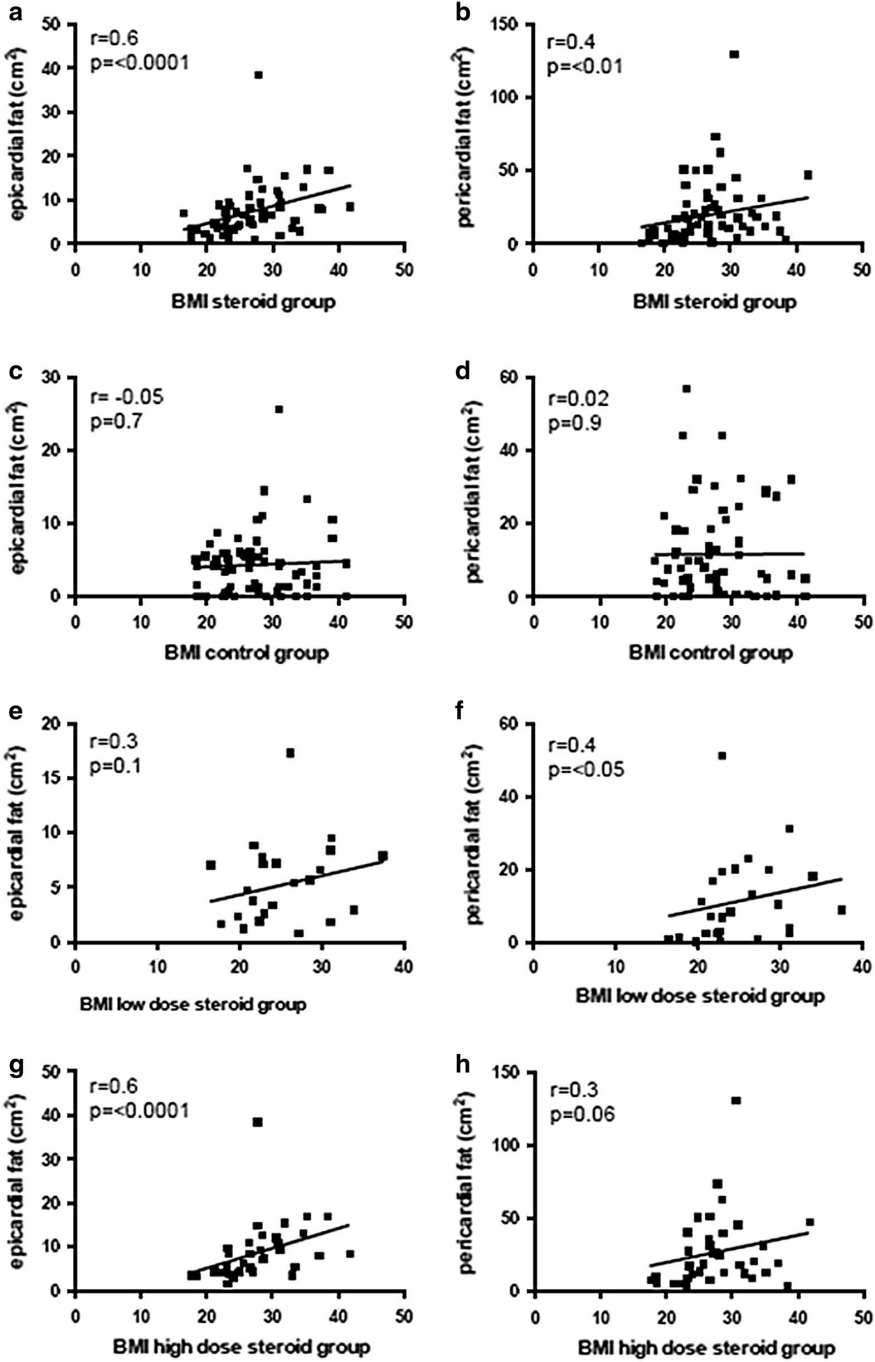
Correlation of multiple parameters in steroid-treated patients and matched steroid-naïve controls. **a**, **b** Epicardial and pericardial fat with BMI in patients with steroid therapy. **c**, **d** Epicardial and pericardial fat in age, sex and BMI matched steroid-naïve controls. **e**, **f** Epicardial and pericardial fat with BMI in the low-dose steroid group (<7.5 mg prednisone equivalent daily). **g**, **h** Epicardial and pericardial fat with BMI in the high-dose steroid group (>7.5 mg prednisone equivalent daily)

We found a correlation between epicardial and pericardial fat and the BMI of patients in the steroid group, see Fig. 3. This holds also true for epicardial fat and BMI in the high-dose steroid group (p < 0.001), as well as for pericardial fat in the low-dose steroid group, p < 0.05. Furthermore, statistical analysis revealed a trend for pericardial fat in the high-dose steroid group to be related to BMI (p = 0.06), and also for epicardial fat in the low-dose steroid group (p = 0.1). No correlation of epicardial or pericardial fat deposition with BMI could be detected in the control group, see Fig. 3.

**Fig. 4:**
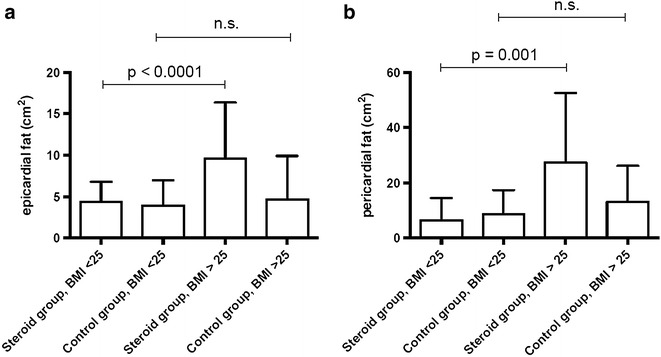
Comparison of epicardial and pericardial fat deposition in steroid-treated patients vs. controls with BMI >25 and BMI <25. **a** Amounts of epicardial fat in steroid-treated patients with BMI >25 (obese) and <25 (non-obese) compared to steroid-naïve controls. **b** Amounts of pericardial fat in steroid-treated patients with BMI >25 and <25 compared to steroid-naïve controls

**Fig. 5:**
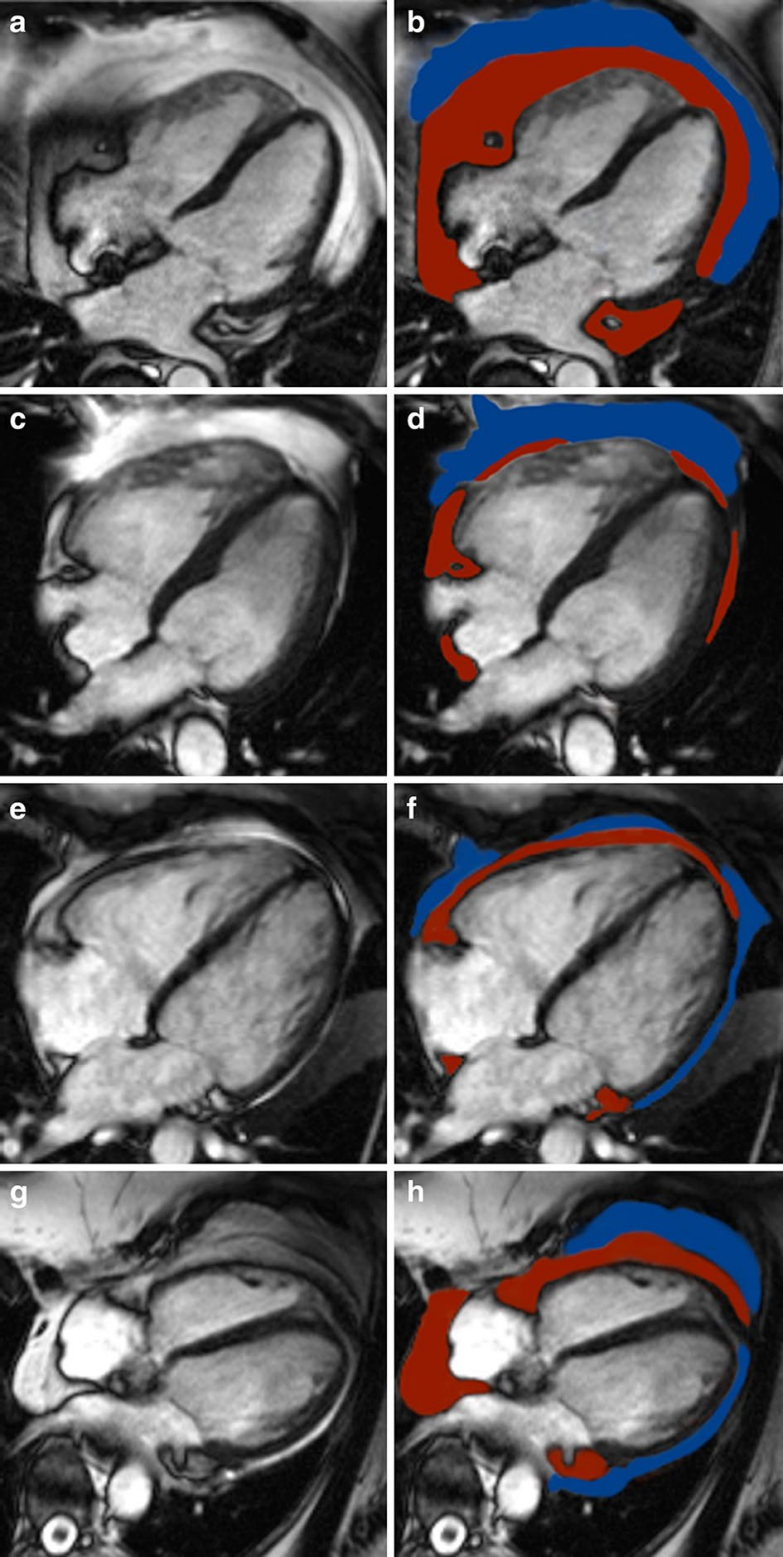
Patient examples of 4-chamber oriented end-diastolic images for determination of epicardial and pericardial fat. **a**, **b** 55-year old male with high-dose steroid treated rheumatoid arthritis for more than 8 years. Patients BMI was 27.8 kg/m^2^, beside arterial hypertension he suffered from diabetes. CMR 4-chamber view revealed extensive epicardial fat deposition (shown in *red*) and pericardial fat deposition (shown in *blue*). **c**, **d** Age, sex and BMI matched steroid-naïve control to the high-dose steroid-treated patient in panels A/B with less amounts of epicardial and pericardial fat. **e**, **f** 49-year old steroid-naïve female (control group) with moderate epi- and pericardial fat deposition despite a high elevated BMI of 41.2 kg/m^2^ (obese class III). **g**, **h** 69-year old steroid-naïve female (control group) with a BMI of 26.8 kg/m^2^. Despite only moderate elevated BMI and no history of steroid intake, this patient showed considerable amounts of epi- and pericardial fat, underlining the lack of association between BMI and amounts of epi- and pericardial fat in the steroid-naïve control group

Dividing patients on steroids and matched controls in an obese (BMI > 25 kg/m^2^) and a non-obese group (BMI < 25 kg/m^2^) revealed, that steroid patients with a BMI > 25 kg/m^2^ showed significantly more epicardial fat than steroid patients with a BMI < 25 kg/m^2^ (p < 0.0001). Similar results could be found for pericardial fat in the steroid-treated group (p = 0.001), see Fig. 4. However, no statistical significant difference in cardiac fat distribution between obese and non-obese control patients could be reported. Typical CMR results are displayed in Fig. 5.

## Discussion

To the best of our knowledge, this is the first study systematically evaluating the impact of long-term steroid use on cardiac fat deposition in patients with different rheumatic disorders.

The main findings of the study are as follows: (1) Long-term steroid use is associated with increased epicardial and pericardial fat deposition compared to steroid-naïve controls. (2) High-dose steroid therapy (>7.5 mg prednisone equivalent daily) leads to higher amounts of epicardial and pericardial adipose tissue compared to low-dose steroid therapy. (3) Cardiac fat distribution correlates with BMI in steroid treated patients, but not in steroid-naïve controls.

Thus, in awareness that cardiac fat may represent a new risk factor for CAD, patients with rheumatic disorders and long-term steroid therapy should be closely monitored for early prevention of cardiovascular diseases.

### Patient population

The mean patient age (54 years) and gender distribution (71 % female) are in line with previously published studies dealing with patients on long-term steroid treatment [18]. As to expect, the steroid group demonstrated more epicardial and pericardial fat than the steroid-naïve control group. Diabetes was present in 10 patients of the steroid group, compared to only one patient in the control (p = 0.01), underscoring the need for glucose-lowering medication in a cumulative dose-dependent manner in those patients [19, 20]. Moreover, the prevalence of arterial hypertension was increased in patients on steroid therapy vs. steroid-naïve controls (33 patients on steroids vs. 23 steroid-naïve controls, p = 0.10). These findings are in line with results from other groups, demonstrating that steroid treatment contributes to development of arterial hypertension [21, 22].

In total 9 patients, all treated with steroids, showed evidence of CAD, which might have several reasons: First, it is known that a high level of cortisol can lead to adverse events including cardiovascular disease mediated by effects which favors arterial hypertension, obesity, diabetes, hypercholesterolemia [23]. Second, it is known that some rheumatic disorders (e.g. rheumatoid arthritis, systemic lupus erythematosus (SLE), ANCA-associated vasculitis) [9], implicate an increased cardiovascular risk profile.

### Low-dose steroid group vs. high-dose steroid group

Several studies suggested a dose-dependent interaction between steroids and the prevalence of adverse effects [13, 24]. Our steroid population was divided in a low-dose (<7.5 mg prednisone equivalent daily) and high-dose steroid group (>7.5 mg prednisone equivalent daily), taking steroids for at least 6 months, which is in line with other reports [13, 24]. Consequently, BMI was higher in the high-dose steroid group (28 ± 6 kg/m^2^) than in the low-dose steroid group (25 ± 5 kg/m^2^, p = 0.05). Interestingly, patients in the high-dose steroid group showed increased prevalence of arterial hypertension and diabetes compared to the steroid-naïve control group. However, this difference was not statistically significant.

Previous studies demonstrated that cardiac fat volumes gradually increased with the number of metabolic syndrome components [25]. Of note, in our population CAD was more prevalent in the high-dose steroid group (n = 7) compared to the low-dose steroid group (n = 2; p = 0.29). This trend supports the hypothesis that high levels of steroids lead to cardiovascular disease mediated by steroid side effects such as arterial hypertension, obesity, diabetes, or hypercholesterolemia [23, 26–28].

### Epicardial and pericardial fat

Steroids are known to cause obesity [23], which is an important component of the metabolic syndrome. Moreover, previous studies could demonstrate that high-dose steroids yield an increase of visceral adipose tissues [29–31].

Epicardial fat surrounds the coronary arteries, and shares embryological origin with abdominal visceral fat, which is known to be an independent cardiovascular risk factor [32]. A frequent discussed mechanism for coronary atherosclerosis are paracrine effects by the close proximity to the coronary arteries and the high content of secreted inflammatory factors in epicardial adipose tissue [33–35].

Patients with high-dose steroid therapy showed significantly more epicardial fat compared to matched steroid-naïve controls. Furthermore, patients on high-dose steroid therapy had significantly increased amounts of epicardial fat compared to patients on low-dose steroid therapy, pointing towards a cumulative dose-dependent effect of steroids on cardiac adipose tissue accumulation. As described above, use of steroids might result in adverse effects, which are similar to the components of the metabolic syndrome. A recent study could show that patients with metabolic syndrome had significantly larger areas of epicardial and pericardial fat in comparison to subjects without metabolic syndrome [3]. In line with these results, our study revealed significantly elevated amounts of epicardial and pericardial adipose tissue in the steroid-treated group compared to steroid-naïve controls. Of note, the results for epicardial fat in our high-dose steroid group 7.2 [4.2–11.1] cm^2^ are in line with the amounts of epicardial fat in this latter study [3]: 8.4 [3.9–17.5] cm^2^. Moreover, the amounts of pericardial fat in our high-dose steroid group 18.6 [8.9–38.2] cm^2^ nicely matches the results of pericardial fat in patients with metabolic syndrome 19.1 [6.2–61.3] cm^2^, strongly suggesting similar effects of a (high-dose) long-term steroid therapy and the metabolic syndrome on cardiac adipose tissue deposition [3, 12].

### Cardiac fat and BMI

In the steroid treated group epicardial and pericardial fat correlated with patients BMI, also see Fig. 3. Looking at patients with high-dose steroid therapy, this holds also true for the correlation of epicardial fat with BMI (p < 0.0001). Moreover, there was a trend for correlation of pericardial fat with BMI in the high-dose steroid group (p = 0.06). In the low-dose steroid group there was also a trend for correlation of epicardial fat and BMI (p = 0.1), whereas pericardial fat correlated with BMI (p < 0.05), suggesting that even in low-dose steroid treated patients weight gain is yielding increased cardiac adipose tissue accumulation.

Dividing steroid treated patients and matched controls in an obese (BMI > 25 kg/m^2^) and a non-obese group (BMI < 25 kg/m^2^) [36] revealed, that patients within the steroid group and a BMI > 25 kg/m^2^ showed significantly more epicardial fat than patients in the steroid group with BMI < 25 kg/m^2^ (p < 0.0001). Similar results could be found for pericardial fat in the steroid group (p = 0.001). However, no correlation of epicardial or pericardial fat deposition with BMI could be detected in the steroid-naïve control group. These findings confirm another large study measuring epicardial fat thickness by computed tomography in 970 patients. In this study epicardial fat was associated with presence of CAD, as well as CAD severity, but not with BMI, supporting our results [37].

### Clinical implications

On the basis of the data presented, it may be safe to assume that patients with rheumatic disorders on long-term steroid therapy suffer from increased cardiac fat deposition compared to steroid-naïve controls. Furthermore, this effect seems to be dose-dependent, resulting in higher amounts of cardiac fat in patients with long-term use of >7.5 mg prednisone equivalent/day. Adding this new additional cardiovascular risk factor to our per se high-risk population for CAD, careful cardiovascular monitoring is mandatory.

Moreover, as stated by the European League Against Rheumatism (EULAR) recommendations on systemic steroid use [38], there should be effort to achieve a maximum of effectiveness with a minimum of toxicity.

Further large, randomized, multi-center studies are needed to clarify if quantification of cardiac fat distribution in long-term steroid treated patients with rheumatic disorders has the potential to serve as a powerful diagnostic tool in detecting patients of additional cardiovascular risk in this per se high-risk population for CAD.

### Limitations

The quantification of epicardial and pericardial volumes in a single CMR four-chamber view instead of covering the whole ventricle by volume quantification in every short-axis slice might be susceptible to bias. However, recent reports demonstrated that this approach shows good correlation to the time-consuming Simpson method. This holds true for CMR imaging as well as for CT imaging [3, 12, 16, 39]. Another limitation might be the lack of standardized reference values for epicardial and pericardial fat volumes, which presently restrict a more widespread use in clinical routine. However, our results in the high-dose steroid group are similar to the results reported in patients with metabolic syndrome [3], suggesting a common pathway between high-dose steroid effects (steroid induced fat distribution) and effects in patients with metabolic syndrome. Moreover, there are different confounders, which must be addressed. First, we cannot exclude confounding by indication, since higher doses of steroids might be prescribed to those patients with more severe course of the disease. Second, patients were not paired-matched for arterial hypertension and CAD. Third, it is ambiguous, whether rheumatic disorders themselves might have impact on the distribution of cardiac adipose tissue. Fourth, our control group was not matched for rheumatic disorders, since a potential deprivation of steroids would have been largely unethical. Thus, in some of the results, it might be difficult to differentiate if they are attributable to steroid use only, to disease severity, or a combination of both.

## Conclusions

The present data suggest that the use of steroids in patients with different rheumatic disorders is associated with increased cardiac adipose tissue accumulation. This effect seems to be stronger in patients with high-dose prednisone equivalent doses (>7.5 mg daily for at least 6 months) than in patients with low-dose prednisone equivalent doses, suggesting a cumulative dose-dependent effect.

## Abbreviations

ANCA: anti-neutrophil cytoplasmic antibody
BMI: body mass index
CAD: coronary artery disease
CMR: cardiovascular magnetic resonance
CT: computed tomography
ECG: electrocardiogram
EDV: end-diastolic volume
EF: ejection fraction
ESV: end-systolic volume
ICC: intra-class correlation coefficient
ICD: implantable cardioverter defibrillator
IQR: interquartile range
LV: left ventricle
SD: standard deviation
SLE: systemic lupus erythematosus
